# 3D Motion Analysis for the Assessment of Dynamic Coupling in Transtibial Prosthetics: A Proof of Concept

**DOI:** 10.1109/OJEMB.2023.3296978

**Published:** 2023-07-19

**Authors:** Sean Cullen, Ruth Mackay, Amir Mohagheghi, Xinli Du

**Affiliations:** Department of Mechanical and Aerospace Engineering, College of Engineering, Design and Physical SciencesBrunel University London 387820 UB8 3PH Uxbridge U.K.; Division of Sport, Health & Exercise Sciences, College of Health, Medicine and Life SciencesBrunel University London 387820 UB8 3PH Uxbridge U.K.

**Keywords:** Dynamic coupling, motion analysis, pistoning, quantitative methods, transtibial prosthetics

## Abstract

Assessment of coupling between transtibial sockets and users is historically based on clinicians’ observations and experience, but can be inaccurate and unreliable. Therefore, we present a proof of concept, for five out of six possible degrees of freedom coupling metric system for a socket, using motion analysis calibrated on a 3D printed limb substitute. The method is compatible with any socket suspension method and does not require prior modifications to the socket. Calibration trials were used to locate the axis of rotation of the knee joint referenced against a marker cluster on the thigh; this allowed for the identification of the limb during test trials despite the entire residuum being obscured from view by the socket. The error in the technique was found to be within 0.7 mm in displacement and 0.7 degrees in rotation, based on the control data. Dynamic testing showed the Inter Quartile Range (IQR) of inter time step variance was <0.5 mm/deg for all metrics. The method can form a basis for objective socket evaluation, improve clinical practice and the quality of life for amputees.

## I. Introduction

The effectiveness of coupling between a biological limb and socket is critical for function and user satisfaction of a lower limb prosthesis [1]. Breakdown in coupling effectiveness is often referred to as “pistoning”, i.e., the vertical displacement of the residuum within the socket during ambulation [2], [3], [4], [5], [6]. Pistoning is indicative of a reduction in socket fit and is normally assessed subjectively with user feedback and clinician experience.

Numerous methods have been explored in the literature to quantify pistoning. These methods can be split into two broad groups: sensors within the socket, and external observation techniques [3]. Internal measurements include the use of embedded sensors in the socket and liner that measure the displacement between socket and liner [7], [8], [9], [10], [11], [12]. These methods often require modifications to the prosthetic components or inclusion at the fabrication stage, hindering the clinical applicability and in some cases function of the prosthesis. Quantitative observation-based techniques include radiological measurements, photographic, and motion analysis [13], [14], [15], [16]. Whilst these methods do not offer the same level of portability, they are non-invasive and do not interfere with the function of the prosthesis. Radiological techniques are further limited to static load testing, due to the relatively small measurement volume of the machinery, despite providing arguably the most accurate pistoning measurement, using precise anatomical data. Similarly, many of the photographic and motion analysis techniques, were limited to static loading, and required transparent check sockets [14], [15]. Childers and Siebert introduced a method using motion analysis to determine the dynamic coupling of a socket during the walking gait, however, their approach required holes to be made in the socket, not only compromising the socket but making it unsuitable for vacuum suspension systems [17]. These restrictions introduce a high cost to testing and have likely hindered the adoption of the techniques into clinical practice for objective assessment of coupling.

Furthermore, the movement of the limb and socket during functional activities is not limited to one specific direction and should be considered non-constrained in all directions and rotations allowing for six degrees of freedom (DoF).

Tang et al. explored the concept of compound displacements and rotations within the socket, using motion analysis for dynamic coupling identification in trans femoral amputees. Due to the limitations in determining the position of the residual limb within the socket, the relative movement between the socket and residuum was assumed to be similar to a sliding ball joint, with four degrees of freedom (4 DoF) [18]. A 4 DoF model was also used by LaPrè et al. for kinematic analysis of transtibial amputees, using a numerical skeletal model to simulate the dynamic coupling behaviour within the socket [19].

In this article the naming structure for coupling metrics in 6DoF:

• Proximal/distal (PD) displacement (pistoning)
• Rotation in the sagittal plane (bell clapping)
• Medial/lateral (ML) displacement
• Anterior/posterior (AP) displacement
• Axial rotation around the tibia
• Rotation in the frontal plane

Whilst each dynamic coupling metric the key factors for consideration are displacements in the PD direction, rotations in the sagittal plane and axial rotations; these are most likely to cause hazards whilst walking along with a sense of disconnection for the user [1]. A deeper understanding and ability to quantify the coupling metrics will inform clinical practice, defining safety and comfort levels; and form the basis of impartial comparisons between prosthetic socket and suspension technologies. Ultimately, advancements in clinical measurement and diagnostic techniques will lead to better amputee quality of life outcomes.

The aim of this research was to identify a suitable method for the assessment of as many (DoF) dynamic coupling of a transtibial prosthesis as possible, using 3D motion analysis. This method would be applicable to all types of transtibial suspension and require no modifications to the socket or liner.

## II. Materials and Methods

**Fig. 1.**
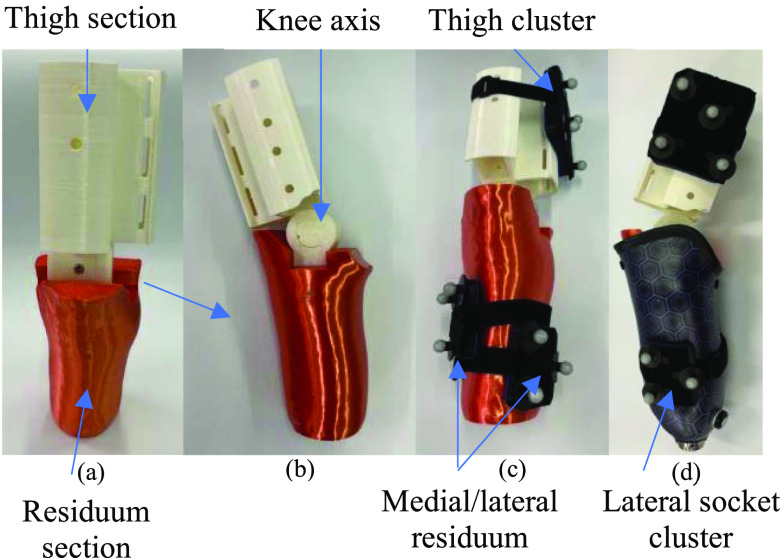
3D printed left lower limb (thigh and shank segments) substitute showing: (a) Anterior view; (b), lateral view; (c) marker (cluster) arrangements for calibration; (d) marker arrangements for trials with the socket.

An experimental setup (socket and limb – Fig. 1) was 3D printed to simulate the coupling interaction. The thigh segment (white) was minimised to reduce the printing time whilst providing mounting points for a marker cluster. The residuum segment (orange) was designed using a 5% reduced scan of a mould from an existing transtibial socket (black and purple), using a predefined method [20]. This reduction allowed for a good fit to the socket with small amounts of movement, replicating the fit of a regular total surface bearing prosthesis. The substitute limb (knee) joint was designed as a (40 mm) revolute joint, allowing for markers to be placed directly onto the joint axis.

Passive infra-red (IR) reflective 14 mm markers were placed as single markers on the medial and lateral ends of the joint axis; a three-marker clusters on the medial and lateral residuum; and a four-marker thigh cluster on the lateral thigh section using rigid plates. Each cluster used a different arrangement of markers to facilitate identification within Qualisys Track Manager (QTM) (Qualisys, Gothenburg Sweden). A 3D IR motion capture system (Qualisys, Gothenburg Sweden) was used consisting of ten Miqus cameras (Qualisys, Gothenburg Sweden) with a 3D resolution of 0.11 mm to capture the marker positions. Sessions were recorded at 120 Hz for compatibility. Plates with 3 markers were required for 6DoF tracking the segments. The thigh plate had 4 markers for easier visual identification, the fourth marker was not used in the data synthesis. The general placement was based on a modified Helen Hayes marker set for the substituted limb sections [21].

### A. Data Collection

**Fig. 2.**
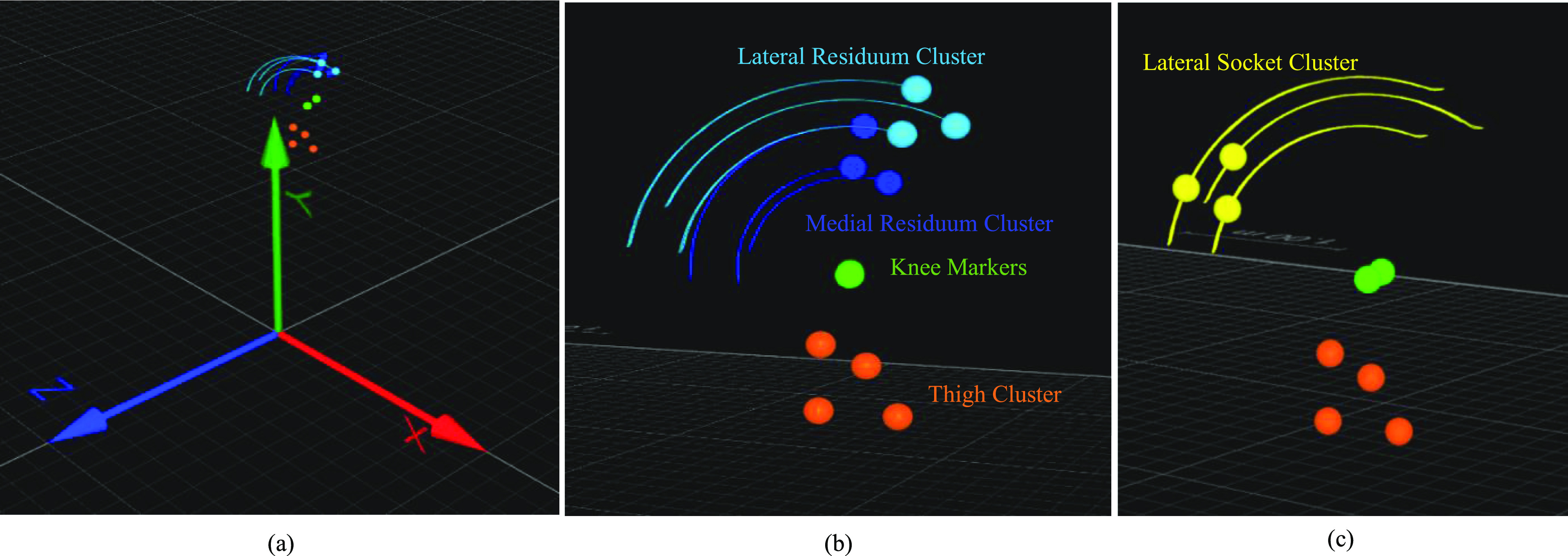
Overview of marker positions for the calibration (a, b) and test trials (c). The limb was orientated with the thigh section at the bottom- and the residuum as the upper-segment: (a) QTM view of marker positions and trajectories for a calibration trial with respect to the global laboratory axes; (b) marker positions viewed from the lateral side of the limb substitute for a calibration trail; (c) a test trial were the residuum marker clusters were removed and a single cluster mounted to the lateral socket.

A series of “calibration” trials were captured, where the limb was held upside-down for the ease of controlling movement, and residuum was outside of the socket and rotated through full range of motion with the thigh section fixed (Fig. 2). Calibration trials allowed identification of the axis of rotation in the substitute limb knee joint [22].

For “test” trials, when the residuum was inside the socket, the residuum clusters were removed, and one lateral three marker cluster was mounted on the transtibial socket donned onto the limb; but the position of the thigh cluster did not alter.

Calibration and test trials were repeated 5 times. A trial was repeated if any missing markers were noted (not including the knee medial and lateral markers during test trials which were expected to be out of the view of the cameras).

### B. Data Analysis

**Fig. 3.**
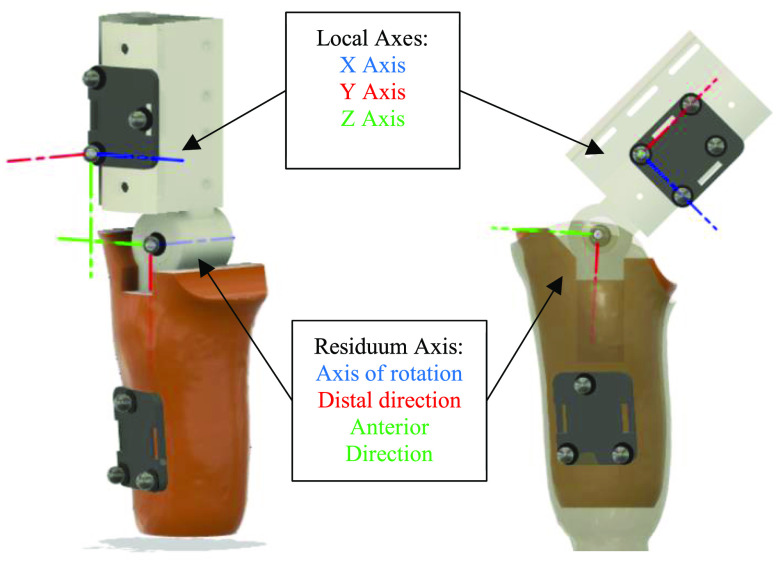
Location and orientation of constructed axes for data point manipulation, shown with and without a semi translucent socket. Marker arrangement indicated for calibration and test trials with high marker plate, lateral residuum/socket marker plates, and the lateral knee marker (CKL equivalent). Note. Calculated Knee Lateral (CKL) is not identified in the figure.

Initially the position of the cluster markers on the thigh segment was defined using the laboratory global origin (centre of the measurement volume – Fig. 2(a)). Thigh cluster markers were then used to create a local co-ordinate system for each time step. The local coordinate system was formed using the line between two vertical thigh cluster markers (local Y axis), the perpendicular line from this line to the third marker (local X axis), and perpendicular to Y and X axes (local Z axis). This was calculated using the cosine and dot product rule for each time step. With the local origin and unit vectors, the position of each marker on the substitute limb was calculated relative to the local co-ordinate system. The position and orientation of the thigh local coordinate system are illustrated in Fig. 3.

The positions of the markers in the local co-ordinate system for the calibration trials were then used to calculate the position and orientation of the substitute limb axis of rotation (representing knee axis of rotation).

The knee joint axis was defined as the line between two hypothetical points representing the epicondyles of femur in a biological limb, Calculated Knee Lateral (CKL) and Calculated Knee Medial (CKM) point. The positions of these points in the local co-ordinate system were determined using an iterative cylindrical best fit model, based on a modified version of the Symmetrical Centre of Rotation Estimation (SCoRE) presented by Ehrig et al. and later developed by Meng et al. [22], [23]. The initial positions of CKL and CKM were based on the markers placed on the medial and lateral sides of the joint axis during calibration trials. The solver reduced the average Root Mean Square (RMS) difference in perpendicular distance of each of the 6 residuum markers to the axis of rotation, across all time steps, by adjusting the position of both hypothetical points.

To isolate movements of the socket relative to the residuum during test trials, an additional residuum co-ordinate system was derived, representing the position and orientation of the residuum. The cardinal axes of this residuum local system comprised of the line connecting CKL to CKM (axis of rotation), the line perpendicular to CKL-CKM in the direction of the centre of the lateral socket marker cluster in the sagittal plane (distal direction axis), and the line perpendicular to these two lines in the anterior direction, with the CKL marker position being the origin, as shown in Fig. 3. As before, the position of each of the 3 socket markers (Fig. 1(d)) in the residuum co-ordinate system was calculated.

**Fig. 4.**
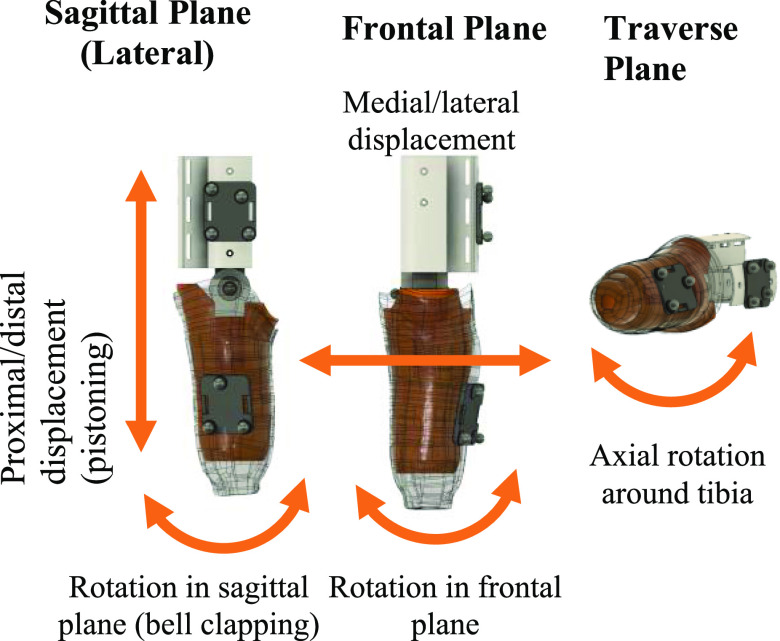
Visual definition of dynamic coupling metrics in principal planes, showing the limb substitute model (orange) within a sample digital socket (translucent grey).

The coupling was defined as the relative displacements and rotations of the socket in the 3 orthogonal directions of the residuum co-ordinate system. The displacement metrics were defined as the change in average distance between the lateral socket markers and a specific axis. The rotation metrics were defined as the rotation of lateral socket markers in a specific plane of movement (transverse, frontal, sagittal), as illustrated in Fig. 4.

It was not possible to isolate the displacements in the AP direction, as the lateral socket marker plate was used to identify the knee rotation angle for the residuum co-ordinate system. The other 5DoF were calculated.

### A. Results

Data collected from the “calibration” trials was used to identify error in the method, as the markers were fixed to the residuum. Therefore, when put through the same mathematical operations as the test data, with the lateral residuum cluster being used over the lateral socket cluster any deviation in the coupling metric values for the markers during “calibration” trials represented a combination of errors from the position of the axis rotation, the measured marker position, and tolerances in the limb substitute joint, as outlined in Table I. One representative calibration trial is reported, as well as the pooled standard deviation as a measure of repeatability between all five trials (Table I).

**TABLE I:** Mean, Errors (Standard Deviation) and Confidence Intervals for the Difference Between Metric Values During the Calibration Trials

Metric	SD	95% CI	Repeatability	
Proximal/Distal Displacement	0±0.18 mm	0.01 mm	0.24 mm	
Rotation sagittal plane	0±0.68 deg	0.04 deg	0.23 deg	
Medial/Lateral Displacement	0±0.68 mm	0.04 mm	0.13 mm	
Axial Rotation around tibia*	0±0.70 deg	0.04 deg	0.13 deg	
Rotation in frontal plane	0±0.21 deg	0.12	deg	0.22 deg

* With anomalies removed.

**Fig. 5.**
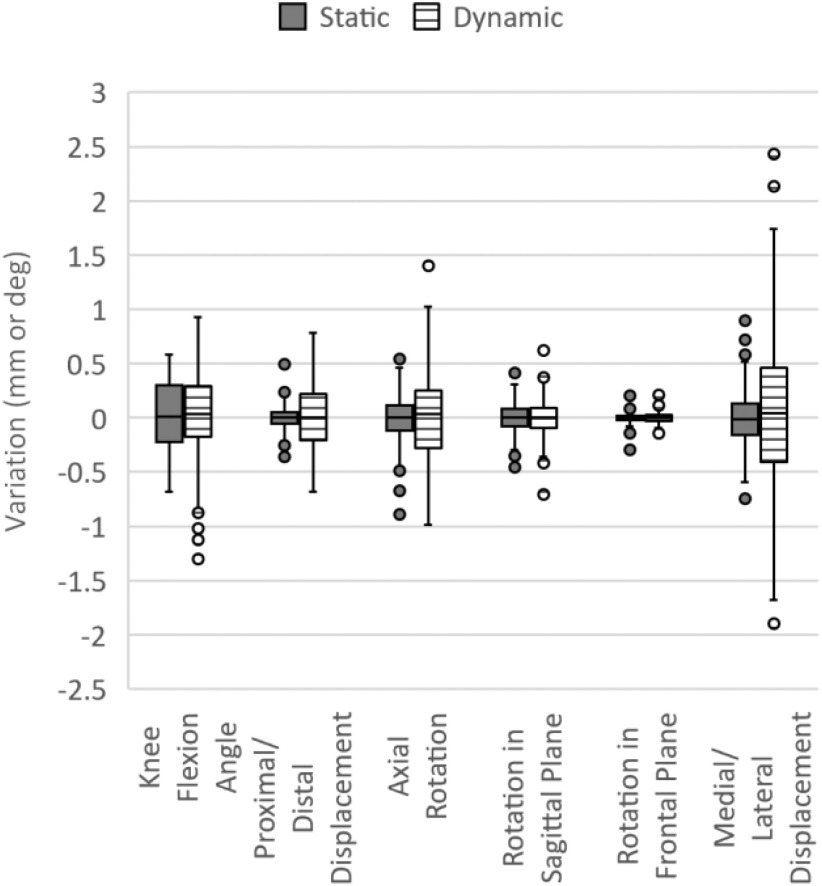
Box plot of variation in metric values between time steps with static tests in block colour and dynamic tests in striped for a single flexion/extension event.

To assess comparative accuracy of the method, flexion tests were conducted with the socket fixed within the laboratory measurement volume (static) and with the limb substitute being moved freely (dynamic) to simulate walking through the measurement volume. The difference in the metric value for each time step for both static and dynamic test trials is shown in Fig. 5. This is a measure of the additional noise introduced by moving the limbs substitute, resulting on continuously changing local axis position and orientation, resembling the effect of movement on measurement accuracy. A single flexion and extension from each static and dynamic trials are compared.

**Fig. 6.**
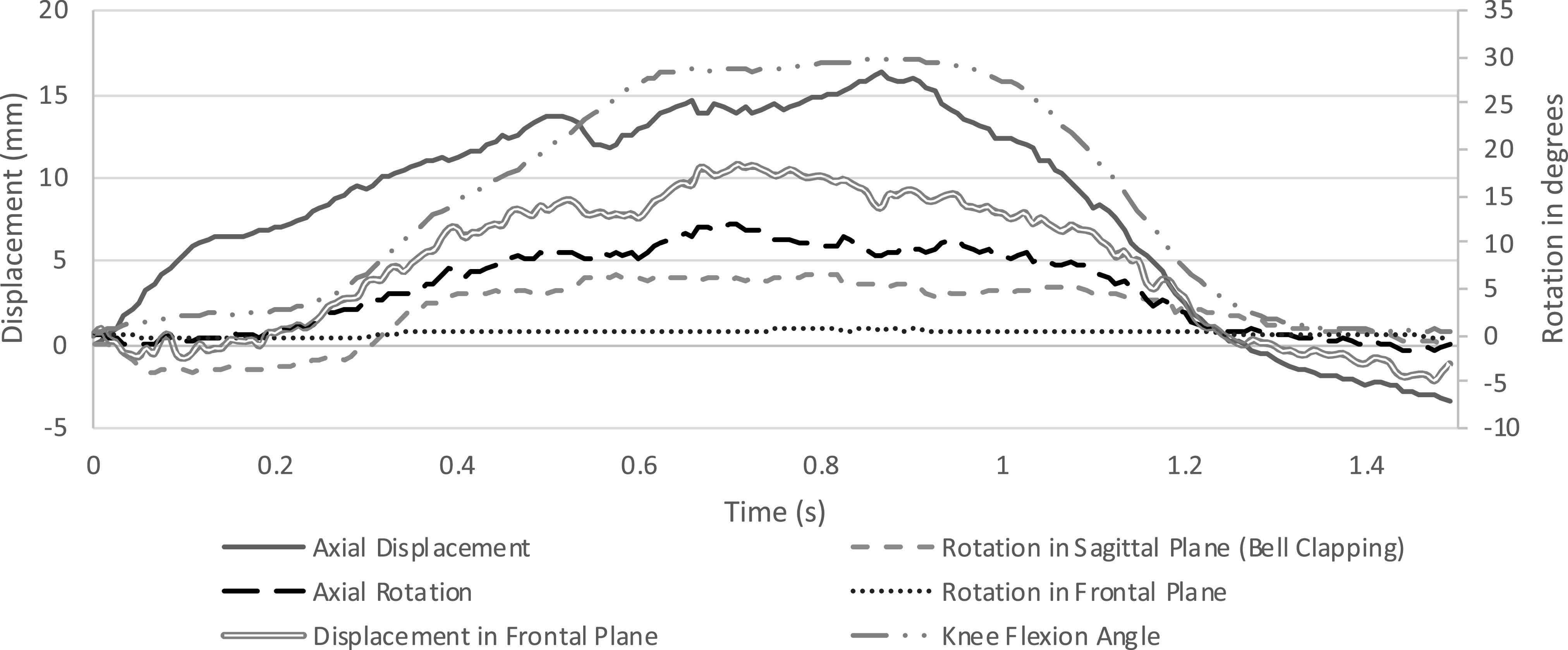
Temporal profile of dynamic coupling metrics for limb substitute model plotted against time for a single flexion-extension event during a test trial (dynamic testing).

Fig. 6 shows temporal profile of data for a representative single flexion-extension of the limb substitute under the dynamic testing conditions. This exemplar profile is not necessarily a representation of the nature of dynamic coupling present in all transtibial sockets during ambulation, but the trends in each metric can be observed over time. For the limb substitute model used, the metrics with the largest total change are PD displacements and rotations in the sagittal plane. For this limb model this is expected as there is no suspension system, and the limb is rigid therefore allowing movements within the socket. During clinical use large changes in coupling metrics could be an indicator of a degeneration in socket fit and or suspension.

## III. Discussion

We presented a method for the identification of dynamic coupling characteristics between socket and residuum for a transtibial prosthesis, without any modifications to the socket. Using a limb substitute, the method was able to identify the following metrics: proximal/distal displacement (pistoning); rotation in the sagittal plane (bell clapping); ML displacement; axial rotation around the tibia; rotation in the frontal plane, based on a 5DoF model. The accuracy of the control data was within 0.7 mm or 0.7 deg (Table I) and the mean inter time step variations was <0.5 mm with dynamic testing (Fig. 5), making the method discussed viable for clinical use.

Unlike other quantitative methods for socket displacement measurements, this method does not rely on imbedded sensors or require modifications to the socket wall, furthermore this method is not limited for use on transparent sockets, as with other motion capture methods [14]. The technique can also make use of a large capture volume and does not restrict the user to walking on a treadmill in close proximity to sensor readers, such as with the method explored by Vempela et al, and could be incorporated into existing clinical spaces for prosthetic fitment [3].

Whilst the equipment cost can be an inhibiting factor for clinical application, the underlying principals are transferable, and could work with newer video-based motion capture systems. As the technology gets more affordable the proposed method could be more widely adopted, increasing the effectiveness of clinical socket evaluations. This technology is new and emerging and require further development for optimal accuracy [24], [25]. Importantly, the method potentially provide a real time dynamic coupling assessment tool for clinicians. Once the calibration data has been captured and calculated the coupling metrics could be outputted in real time.

Using calibration data, the method presented achieved a standard deviation of <0.7 mm for displacements and <0.8 degree for rotations. This is consistent with the results achieved in the literature for other marker based systems, ranging 0.3–2 mm for displacements and 0.2–0.7 degrees for rotations [15], [16], [17], [18]. By contrast X-ray methods achieved a SD of 10–30 mm, and the electromagnetic motion capture system achieved an RMS error of 2 mm, in optimal conditions [3], [26]. For the internally mounted sensor methods, such as the ferrous liner or optical sensor in the distal end of the socket, the achieved RMS accuracies were <3% and <1.95% respectively [8], [9]. It is worth noting however that the method presented also was able to determine the highest number of degrees of freedom, with some methods being limited to PD displacements (pistoning) only. Furthermore, there were no modifications needed to the prosthetic components, and is compatible with all suspension systems, highlighting a clear clinical advantage to the other methods explored to date.

The ideal model for a limb and socket, as discussed would be 6 DoF, however the anterior/posterior direction could not be isolated. The residuum was entirely hidden by the socket and it was not possible to identify the knee flexion angle without using socket markers. Therefore, any displacements in the AP direction between the residuum and socket would be observed as knee flexion/extension. In contrast, the uncertainty introduced by the unmodeled motion had a smaller effect on the measurements than the equivalent 4 DoF models, which have the uncertainty of 2 DoF distributed amongst the measured metrics. Additionally, there is no standard practice on what references points should be used for measuring the coupling metrics, such as distal or proximal end of the tibia, so the specific values for rotations may differ from other studies.

A noteworthy limitation of this method is that any movement in the thigh marker plate/cluster relative to the skin would require recalibration of the model data, as the residuum axis is directly mapped to the local coordinate system described by the thigh marker cluster. Additionally, artifacts introduced by skin movement on a biological limb should be considered and accounted for in experimentation involving animal or human participants, particularly given the residuum axis is mapped, compounding the position errors. A rigid model was chosen for this study to negate these artifacts. For amputee participants artifacts may be increased further by silicone sleeves or other suspension components. For conventional motion analysis, the knee joint centre/axis can be determined more accurately during walking trials using a combination of both the tibia and femur marker segments, reducing the impact of skin artefacts [27], [28]. For prosthetic applications this cannot be used as the residuum position is not fixed within the socket, therefore markers on the socket do not correspond to the true position of the residuum and knee. The rigid marker plates used reduced inter marker variation when compared to being placed on the skin but are still able to move with the skin on the thigh. Additional markers placed on the thigh or other anatomical features such as the hip may help to constrain movement, but the magnitude of the error is unknown. Given the intended clinical application of this method markers affixed to the bones via invasive pins would not be recommended as have been explored in the literature [29]. Therefore, further studies are needed to determine how to reduce errors associated with skin artefacts, and find an alternative method(s) (e.g., a surrogate technique) to estimate dynamic coupling metrics using marker sets and computational methods employed in the present study, which is an ongoing challenge for motion capture [30].

The method presented is accessible to both academia and clinical use, as it is compatible with any 3D motion capture system. In addition, the method does not require precise placement of the markers/clusters, reducing the skill requirements. The marker arrangement used is compatible with a Helen Hayes marker arrangement, so could be used in conjunction to measure other gait metrics [21]. Within each cluster used, the arrangement of markers was unique to help automatic identification, however it does not affect the measurement, so any 3 cluster arrangement can be used.

The arthrokinematic behaviour in biological knees, combined with the varying geometry of contact surfaces result in transitional axis of rotation during flexion and extension [31]. Whilst not relevant for the limb substitute used which has a singular axis of rotation, the authors did explore incorporating a moving axis of rotation model based on knee angle. A weighted cylinder best fit model was used and the position of the axis was calculated for ten flexion angles. Identifying an equation for approximating the instantaneous axis of rotation, more closely modelling the behaviour of a biological knee [32]. This could be implemented for tests involving human participants.

Whilst suspension systems have a critical role in the dynamic coupling behaviour this article uses a suspension less limbs substitute that is held into the socket by its rigid shape. The results shown are the representative of the total amount of coupling dissociation, including both socket fit and suspension components. In addition, the method could be used to evaluate soft residuum substitutes that more closely replicate the residuum – socket behaviour with larger range of motion. The rigid model used in this study replicated a total surface bearing (TSB) fit within the socket, and so may have a different response to flexion and loading than other styles such as patella tendon bearing (PTB).

It is well documented that pistoning, usually referring to generalised dynamic coupling, is associated with decreased socket fit, user comfort, predisposition to falls, and skin damage, especially when considering user feedback [2], [3], [4], [5], [6]. However, to the best of our knowledge there has not been a clinical trial on the acceptable limits and the impact they have on socket fitment, in part due to difficulty of objective measurement. Therefore, the method presented can be used to improve clinical practice, influence the design, and guide adjustment of prosthetic sockets; ultimately leading to a better quality of life for amputees.

## IV. Conclusion

We provided a framework for using motion analysis as a quantitative tool to assess the in situ dynamic coupling metrics in 5 DoF for a fitted transtibial socket, without the requirement for any modifications, achieving an accuracy within 0.7 mm or 0.7 deg, based on the calibration trials. Whilst there are no defined clinical limits on acceptable socket fitment, increased dynamic coupling metrics for a given socket indicate a decline in the fit, and associated comfort experienced by the user.

This study utilised a limb substitute and therefore further study should be conducted into the applicability of this method on biological limbs. Making considerations for the increased artifacts from soft tissue movement while loaded during gait, and translational axis of rotation of the knee joint which will introduce new sources of error which should be considered.

The present work forms a basis for objective in situ clinical socket fitment assessments, with the aim of improving the experience of transtibial amputees, and influencing trends in socket shapes and designs in the future.
